# First Report of Direct Damage Caused by the Stubby-Root Nematode, *Nanidorus minor*, to Strawberry (*Fragaria* x *ananassa*), in Florida

**DOI:** 10.2478/jofnem-2023-0016

**Published:** 2023-06-05

**Authors:** Clemen J. Oliveira, Renato N. Inserra, Johan A. Desaeger

**Affiliations:** Department of Entomology and Nematology, Gulf Coast Research and Education Center, University of Florida, Wimauma, FL, 33598, USA.; Florida Department of Agriculture and Consumer Services, DPI, Nematology Section, P.O. Box 147100, Gainesville, FL 32614-7100, USA.

**Keywords:** Fumigated soil, host-parasitic relationship, nematode parasite of strawberry, nematode population densities, root symptoms

## Abstract

In 2019–2022, declining symptoms were observed in two commercial strawberry farms in Hillsborough County, Florida. The fields in the two farms consisted of raised beds covered by plastic mulch. Both were fumigated with a mixture of 1,3-dichloropropene (40%) + chloropicrin (60%) before planting. Samples collected from large patches with declining plants were infested with stubby-root nematodes. No sting and root-knot nematode species were detected. The results of morphological and molecular analyses indicated that the stubby-root nematode populations were representative of the species *Nanidorus minor*. The two cultivars ‘Florida Brilliance’ and ‘Florida Sensation’ in the two fields included plants with stubby root symptoms showing a reduction in the size of the root system and arrested growth and elongation of the feeder roots on the first strawberry crop. The nematode population densities in the two fields increased at the end of strawberry season and averaged 66 and 96 specimens in 200 cm^3^ soil. In one of the fields, a second strawberry crop was established as in the previous year using the same practices (fumigation and raised beds covered with plastic). However, in this field the population of *N. minor* declined and did not reach damaging levels at the end of the season on the second strawberry crop. The factors causing the decline of the nematode population were not elucidated. This is the first report of a direct damaging effect of *N. minor* to strawberry.

In Florida, strawberry production is an important component of the vegetable industry with an annual value of $282 million (USDA-NASS, 2017). Many factors negatively affect strawberry production in the state, such as diseases and pests, including plant-parasitic nematodes (PPN). The most damaging PPN species to strawberry in order of economic importance are sting nematodes (*Belonolaimus longicaudatus* Rau, 1958) and root-knot nematode species in the genus *Meloidogyne* (Göldi, 1887) Chitwood, 1949. Among these species, *M. hapla* Chitwood, 1949 is the most common because it was introduced into Florida fields with nematode-infested propagative runners (Desaeger, 2019). Although association of stubby-root nematodes, such as *Nanidorus minor* (Colbran, 1956) Siddiqi, 1980 with strawberry (*Fragaria* x *ananassa* Duchesne) has been reported by Rhode and Jenkins (1957), there are no records of infestations of stubby-root nematode species (Trichodoridae (Thorne, 1955) Clark, 1961) causing damage to strawberry in the literature. In 2020, symptoms of decline, consisting of stunted plants showing reduced root systems with a low number of short feeder roots, were observed in a field where preliminary analyses of a few samples indicated that stubby-root nematode species were the predominant PPN. Infestations of stubby-root nematodes are common in Florida fumigated fields (especially tomato and strawberry fields) because these nematodes are more tolerant to currently used fumigants, or because they escape the effects of the fumigants by migrating into the deep layers of the soil not reached by the available fumigants after the loss of the methyl bromide (Weingartner and Shumaker, 1990; Weingartner et al., 1993). A field investigation was conducted with the objectives to determine: i) the involvement of stubby-root nematode species in the crop decline, ii) the nematode species inducing the decline, and iii) the symptoms induced on strawberry by the nematode infestation.

Two fumigated farms located in the same strawberry producing areas of Central Florida were selected for this study and indicated as Farm 1 and 2 for convenience. In Farm 1, where the first declining symptoms were noticed, certified runners of the strawberry cultivar ‘Florida Brilliance,’ imported from a nursery in Quebec, Canada, were transplanted in October 2019, in raised beds covered by a totally impermeable field plastic (TIF Total Blockade, Berry Plastics Corporation, Evansville, Indiana) after fumigation with a mixture of 1,3-dichloropropene (40 %) + chloropicrin (60 %) (PicClor60 @ 336 kg/ha). In this farm, observations were conducted on two strawberry crops, the first in 2020 and the second in 2021. In Farm 2, certified runners of the strawberry cultivar ‘Florida Sensation,’ imported from a nursery in California, were transplanted in October 2021 in raised beds covered by a totally impermeable field plastic after fumigation with the same fumigant. In this farm, the observations were conducted only on one strawberry crop in 2022. Soil and root samples were collected from the two farms at different time intervals as specified below. Plant-parasitic nematodes were extracted from 200 cm^3^ soil using a modified Baermann funnel technique (Rodríguez-Kábana and Pope, 1981) and identified using a compound microscope. Specimens were hand-picked in tap water, immobilized by gentle heating, and mounted in water agar on a slide for measurements and photographs using a modified technique described by Esser (1986). Measurements of specimens were made using a Nikon (Optiphot) ocular micrometer. Additional specimens were sent to Dr. Sergei Subbotin for sequencing and phylogenetic analysis to confirm our morphological identification. Root symptoms were examined, and photographs were taken using a digital microscope Keyence (VHX-7000 series, KEYENCE CORPORATION, Itasca, Illinois).

In Farm 1, the declining symptoms were observed five months after transplanting (March 16, 2020), at the end of the season of the first strawberry crop, when soil and root samples, for a total of 17, were collected. In 2021, after soil fumigation and planting of the runners of second strawberry crop, another three and three samples of soil mixed with roots were collected at midseason (January 2021) and the end of the season (March 2021) of the second strawberry crop, respectively. The aim of this sampling was to verify the persistence of the nematodes in the field and the reoccurrence of symptoms they caused on the second strawberry crop. In Farm 2, a total of two and five samples were collected as mentioned above from one strawberry crop only, at midseason (January 2022), and the end of the season (March 2022), respectively. Nematode populations extracted from the samples collected in the two farms consisted mainly of stubby-root nematodes. No root-knot and sting nematodes were found associated with the stubby-root nematodes. The morphology of the stubby-root nematodes fitted that of *Nanidorus minor* Siddiqi 1980. The morphological characters of eight females of this population included: Body length (L) = 700.9 ± 78.8 μm (619.1 − 769); Stylet length (ST) = 34.1 ± 1.1 μm (32.2–35.6); Body length/greatest body diameter (a) = 17 ± 1.3 μm (15.5–19.8); Body length/distance from anterior end to posterior end of median pharyngeal bulb (b) = 5.1 ± 0.3 (4.8–5.6); Distance of anterior body end from the vulva/body length % (V) = 51.7 ± 1 (50–53.5). A phylogenetic analysis using the D2-D3 expansion fragments of 28S rRNA gene with 58 valid and putative species of stubby root nematode revealed that the strawberry population collected in Hillsborough County was 99.18 to 100% identical to other populations of *N. minor* (Subbotin et al. 2020). The results of the morphological analysis were confirmed by those of the phylogenetic analysis that were published by Subbotin et al. (2020).

In Farm 1, the average number of stubby-root nematodes collected at the end of the season of the first strawberry crop, in 2020 was 96/200 cm^3^ soil. These population densities were associated with stunted plants showing reduced root systems with a low number of short-feeder roots, consistent with stubby-root symptoms like those described for *N. minor* by Christie and Perry (1951) on other crops (Figure 1). Stunted plants were localized in large areas (8.1 meter wide and 13.7 meter long) in the field. The populations densities detected for the second strawberry crop at the mid- and end of season in 2021 were lower than those in the previous crop in 2020, and averaged 7 and 4/200 cm^3^ soil, respectively. No reoccurrence of the stunting symptoms was noticed on the second strawberry crop at these nematode densities. These findings indicated the populations of *N. minor* did not increase to damaging level during the second strawberry cycle.

**Figure 1:**
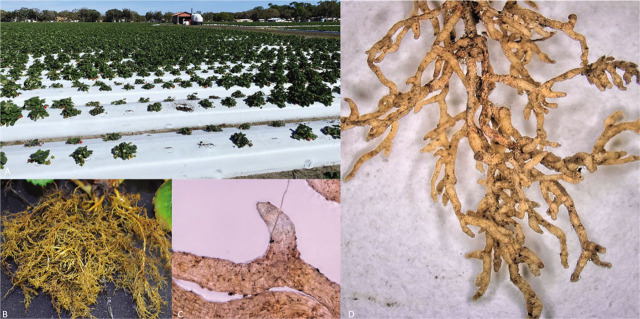
Strawberry damage caused by *N. minor*. (A) Plant stunting and canopy reduction observed in a commercial strawberry field; (B) Stubby root in strawberry; (C) Digital microscopy (magnification 200x) of a section of a parasitized strawberry root tip; (D) Digital microscopy of a parasitized strawberry root system (magnification 20x).

In Farm 2, an average of 10 and 66 specimens/200cm^3^ soil was found in January 2022 and March 2022, respectively. In this field, the population density increased at the end of the 2021–2022 season, resulting in visual field damage localized in large areas (13.8 meter wide and 17.4 meter long) as observed in Farm 1. In both farms, strawberry root damage occurred at the end of the season when the highest population densities were recorded.

The results of this field study provide evidence that *N. minor* can induce serious damage to strawberry comparable to that reported in fields infested with sting nematodes. However, in contrast to observations on sting nematodes, the populations of this species did not increase consistently on the second strawberry crop. Data in our study are not sufficient to explain the decline of *N. minor* populations on the second strawberry crop cycle. We can hypothesize that *N. minor* populations localized in the top layers of the soil during the second strawberry cycle were more vulnerable to the adverse effect of the second application of the fumigant, which prevented nematode reproduction.
